# Abnormal miR-122-5p expression in decidual NK cells and its impact on trophoblast behavior: insights into unexplained recurrent pregnancy loss

**DOI:** 10.7150/ijms.101865

**Published:** 2024-10-28

**Authors:** Yuan Liu, Liman Li, Ting Feng, Wenjie Zhou, Ying Liu, Yueli Mu, Zhuoxu He, Hong Li

**Affiliations:** 1 Center of Translational Medicine, Key Laboratory of Birth Defects and Related Diseases of Women and Children of Ministry of Education, West China Second University Hospital, Sichuan University, Chengdu, 610041, China.; 2 Department of Andrology/Sichuan Human Sperm Bank, West China Second University Hospital, Sichuan University, Chengdu, 610041, China.

**Keywords:** recurrent pregnancy loss, maternal-fetal interface, decidual natural killer cells, miR-122-5p, trophoblast

## Abstract

In the early stages of pregnancy, the maternal-fetal interface is enriched with natural killer (NK) cells that release growth factors to support fetal development and promote the remodeling of uterine spiral arteries. Previous studies have shown that the aberrant frequency and activity of decidual natural killer (dNK) cells are associated with recurrent pregnancy loss (RPL). Various factors regulate the roles of dNK cells and their interactions with trophoblasts to facilitate the colonization and maturation of semiallogeneic embryos. However, knowing precise molecular mechanisms involved in this requires further investigation. Earlier studies revealed that microRNAs (miRNAs) play a significant role in regulating the functions of decidual stromal and trophoblast cells. Although there are few studies on the intervention of malfunctioning dNK cells, this strategy shows promise in regulating abnormal miRNA production in NK cells. This study confirmed miR-122-5p downregulation in dNK cells from patients experiencing unexplained RPL. miR-122-5p regulates apoptosis, inflammatory factor secretion, and cytotoxicity of NK cells. miR-122-5p may contribute to immune tolerance at the maternal-fetal interface by targeting transcription factor T-bet. This study provides a deeper understanding of the mechanisms by which miR-122-5p regulates the function of dNK cells and trophoblasts at the maternal-fetal interface to ensure successful pregnancy.

## Introduction

Recurrent pregnancy loss (RPL) is defined as the occurrence of two or more pregnancy losses before 24 weeks of pregnancy, causing significant sorrow to many families globally. Fifty percent of RPL instances lacking a definitive reason were categorized as unexplained RPL (URPL) 1. The prevention and treatment of RPL pose a pressing challenge for medical professionals specializing in reproductive medicine. Pregnancy is a unique immune phenomenon wherein the mother coexists harmoniously with the semiallogenic fetus 2. Decidual immune cells (DICs), predominantly decidual natural killer (dNK) cells (approximately 70%), T cells, macrophages, and dendritic cells, are abundant at the maternal-fetal interface. Their interactions with trophoblast cells produced from the conceptus efficiently inhibit harmful immune responses to paternally transmitted alloantigens and confer unique features that differ from their counterparts in peripheral blood 3-5. These cells exhibit intense residency, secrete numerous cytokines, angiogenic factors, and growth factors, and play crucial roles in decidual transformation, uterine vascular adaptation, and process of placentation 6-9. DICs play a vital role in establishing the ability to receive, initiating pregnancy at the embryonic stage, and producing growth factors necessary for maintaining the fetus. Multiple studies have suggested that deficits or abnormal activation states in dNK cells contribute to pathophysiological processes leading to unfavorable prenatal outcomes and pregnancy complications 9-11. The potential of diagnosing and treating aberrant dNK cells shows promise despite the lack of research in this field.

MicroRNAs (miRNAs) are tiny noncoding RNAs with a negative regulatory role in gene expression and have been implicated in the development and advancement of many diseases 12. More than 400 miRNAs have been identified in NK cells, indicating their involvement in regulating cellular function 13. Cichocki *et al.* revealed that miR-181 expression significantly affects the development of human NK cells from CD34+ hematopoietic progenitor cells and the production of primary CD56+ NK cell-derived interferon-γ (IFN-γ) 14. Increased miR-146a expression has been observed in NK cells of patients with chronic hepatitis B and hepatocellular carcinoma (HCC) 15. miR-362-5p also improves the performance of NK cells by specifically targeting cylindromatosis, which negatively regulates the nuclear factor-κB signaling pathway. Therefore, manipulating NK cell function by modulating miRNA expression represents a potential strategy for modulating the local immune microenvironment.

This study revealed a substantial decrease in miR-122-5p expression in dNK cells from patients with URPL. The regulatory impact of miR-122-5p on NK cells was determined via *in vitro* experiments, finally revealing the existence of the miR-122-5p/T-bet/IFN-γ pathway in NK cells, which might have a crucial function at the interface between the mother and the fetus.

## Materials and methods

### Specimen collection and ethical permission

Decidua samples from the healthy control (n = 13, HC) group and the unexplained pregnancy loss (n = 15, URPL) group were collected from the West China Second Hospital of Sichuan University from November 2021 to May 2022 (baseline characteristics of the population are listed in Supplementary Table S1). The exclusion criteria included pregnant women with (a) autoimmune disease; (b) chromosomal diseases such as gene-linked defection and abnormal embryo chromosomes; (c) bacterial, fungal, or viral infection such as hepatitis B or syphilis; (d) abnormalities of physiological structures such as genital tract malformations; (e) abnormalities in endocrine hormone levels; and (f) organ transplantation or blood transfusion. Fresh tissue samples were collected under aseptic conditions and transferred to the laboratory for processing as soon as possible. Peripheral blood mononuclear cells (PBMCs) for NK cell expansion were collected from 10 healthy women of childbearing age; the exclusion criteria for volunteers were consistent with those described above. The Ethics Committee of West China Second Hospital of Sichuan University approved the study protocol [Approval number: Medical Research 2020 (029)]. All human specimens were processed in a biosafety cabinet following the guidelines of West China Second Hospital of Sichuan University.

### Isolation of immunocytes from decidua and peripheral blood

Human decidual tissue was collected from the operating room, and the appropriate phosphate buffer solution (PBS) containing penicillin-streptomycin (PS, Gibco, USA) was added. After cleaning with PBS, surgical scissors were cut into tissue blocks of 1-3 mm^3^. Then, 1 mg/ mL collagenase IV (Sigma, United States) and 150 U/mL DNase I (Applichem, Germany) were added to a total volume of 10 mL for 40 min at 37 ℃ with gentle agitation. The 2 mL fetal bovine serum (FBS, Hyclone, USA) was used to terminate the digestion, and the suspension was filtered successively through 100 μm and 70 μm filters. The filtered suspension was centrifuged at 500×g for 10 min, the upper liquid was discarded, and the lower cells were resuspended in a complete medium. Percoll solution (Pharmacia, Sweden) with a concentration gradient of 20%, 40%, and 60% were prepared and successively added to the centrifuge tube. The suspension was added to the upper layer and centrifuged at 500×g for 20 min to draw 40-60% white membrane layer cells.

Density centrifugation was performed to isolate PBMCs using Ficoll-Hypaque (TBD Science, Tianjin, China) according to the manufacturer's protocol. Peripheral blood supplemented with heparin lithium was diluted with an appropriate volume of PBS. Second, the same volume of Ficoll gradient was used, and the mixture was centrifuged at 500×g for 20 min. A pasteur pipette was used to harvest the PBMCs, after which the PBMCs were washed with PBS.

### Flow cytometric analysis

Cells were washed and prepared as a single-cell suspension at a concentration of 2×10^6^ cells/mL. Then, 100 μL aliquots of the samples were added to a round-bottom tube (Becton Dickinson, USA). Next, dead/live cells were stained using the Zombie Aqua fixable viability kit (BioLegend, USA) and then blocked with 5 μL Fc-Block (BioLegend, USA) at room temperature for 30 min. For the intracellular cytokines, 2 μL Cell Activation Cocktail (BioLegend, USA), brefeldin A (BioLegend, USA), and monensin (BioLegend, USA) be added to each round bottom tube for incubation at 37°C for 4 h. Before the staining of intracellular cytokines and lytic granules, the cells had been fixed and permeabilized for 30 min. The 3 μL monoclonal antibody was added and incubated at room temperature for 30 min. The gating strategy is shown in supplementary Figure S1, and all the antibodies used are listed in Table 1**.**

### Cell culture

PBMCs for NK cell expansion were collected from the peripheral blood of healthy fertile female volunteers as described previously. We utilized a natural killer cell culture kit plus (NK-KITP, DAKEWE) to perform the NK cell culture. The process involved adding a medium and recording the NK cell purity according to the manufacturer's protocol. The cells were cultured in 6-well plates at an initial density of 1.5×10^6^/mL in a humid atmosphere with 5% CO_2_ at 37°C. To induce dNK-like cells, TGF-β1 (5 ng/mL, 240-B, R&D, USA), hCG (15 IU/mL, Lizhu, Zhuhai, China), and IL-15 (10 ng/mL, AF-200-15, PeproTech, USA) were added to the medium on the 7th, 9th, and 11th days of the process. At the end of day 14, the cells were harvested, and negative selection was performed based on the purity of the cultured NK cells.

The K562 cells and HTR-8/SVneo cell lines (Shanghai Institutes of Biological Sciences, Chinese Academy of Sciences, China) were grown and maintained in RPMI-1640 medium supplemented with 10% FBS and 1% PS (Gibco, USA) in a 5% CO_2_ humid atmosphere at 37°C. Medium replacement and cell passage were performed according to cell density observed under the microscope.

### Purification of NK cells

NK cells were purified via negative selection with a magnetic-activated cell sorter (MACS, Miltenyi Biotec) according to the manufacturer's protocol. The purity of the NK cell population obtained was over 90%, as confirmed by flow cytometry.

### Transfection of NK cells

NK cells were transfected with miR-122-5p mimic (double-stranded RNA), inhibitor (single-stranded RNA), and their negative control (Ribo, Guangzhou) using RiboFECT™CP or Celetrix (DAKEWE, 560 V, 10 ms). Then, the electroporated NK cells were subjected to further experimentation after a rest period of 24 hours. A dead cell removal kit (Miltenyi Biotechnology Incorporation, Germany) was used to remove the dead cells according to the manufacturer's instructions.

### Proliferation potential assay

The proliferation potential of NK cells was analyzed by Ki-67 staining. 2 mL of pre-cooled eBioscience^TM^ Fixation Permeabilization (BD Biosciences) was added into NK cells and incubated for 1 h. The cells were washed with PBS and resuspended in 100 μL PBS. Then, 2 μL of anti-human Ki-67 antibody was added and incubated for 1 h. The cells were resuspended in 100 μL of PBS. The percentage of Ki-67+ NK cells was analyzed using flow cytometry.

### Apoptosis assay

The apoptosis of the transfected NK cells was evaluated via Annexin V staining. Annexin V (2.5 μL) and 7-AAD (1.5 μL) antibodies were added to the resuspended cells (1×10^6^/mL, 100 μL) for 30 min. Then, 300 μL of binding buffer (BioLegend, USA) was added to the stained NK cells, and the percentage of apoptotic cells (Annexin V+) was determined using flow cytometry.

### Cytotoxicity assay

First, transfected NK cells (1×10^6^/mL) were cocultured with targeted tumor cells (K562) at an effector-to-target (E: T) ratio of 10:1 in 200 µL of relevant culture medium in a 5% CO_2_ at 37°C for 4 h. After centrifuging at 300×g for 10 min, the cells were resuspended in 100 µL PBS. The degranulation of NK cells was assessed by CD107a staining. Dead K562 cells were analyzed by PI staining, while Dioc was used to stain NK cells.

### ELISA

Transfected NK cells from different treatment groups were centrifuged at 300×g for 10 min to harvest the supernatant. The secretion of IFN-γ, TNF-α, IL-17A, IL-10, granzyme B, perforin, and granulysin in the medium supernatants was measured with ELISA kits (Ruixinbio Quanzhou, China) following the manufacturer's protocol.

### RNA isolation, cDNA synthesis, and RT-qPCR

Total RNA was isolated from cells using TRIzol reagent (Invitrogen, USA) under RNAase-free conditions as per the manufacturer's protocol. Reverse transcription of the miRNAs was performed using the All-in-One™ miRNA First-Strand cDNA Synthesis Kit (GeneCopoeia, USA). An All-in-One™ miRNA RT-qPCR Detection Kit (GeneCopoeia, USA) was used to quantify the expression of miR-122-5p (HmiRQP0274). Reverse transcription of mRNAs was completed using an Evo M-MLV RT kit with gDNA removed with a qPCR II kit (AG111728, Accurate Biotechnology, Hunan, China). HsnoRNA-U48 (HmiRQP9021) and GAPDH served as controls. RT-qPCR was carried out using 2×SYBR^®^Green Pro Taq HS Premix (AG11701, Accurate Biotechnology, Hunan, China), and the results were analyzed via the qTOWER3G Analytik system (Jena, Germany). The sequences of primer used are listed in Table 2.

### Dual-luciferase reporter assay

A luciferase reporter assay was used to confirm the binding sites for hsa-miR-122-5p in the 3' UTR of *T-bet* mRNA. The *T-bet* 3' UTR of the wildtype and mutant sequence was inserted into the pmiR-RB-REPORTTM (Ruibo, Guangzhou, China), and activated NK cells harboring the mutant or wild-type 3' UTR were cotransfected with the hsa-miR-122-5p or NC mimic by electroporation. Transfected NK cells were collected after a 24-hour rest period, and the supernatant was discarded after centrifugation at 300×g for 10 min. 200 µL of lysate was added and centrifuged at 5000×g for 5 min. 100µL of the collected supernatant was added to the microplate, and then the equal volume of firefly and renilla luciferase detection reagent was added in turn to gently mix. Finally, firefly and renilla luciferase activities were measured using the Dual-Glo Luciferase Assay System (Promega, Italy).

### Migration and invasion assays

The trophoblast cell line HTR-8/SVneo was cultured in RPMI medium supplemented with 10% FBS and 1% PS. The cells were grown in a 5% CO_2_ humidified atmosphere at 37°C. Transwell plates with a filter membrane aperture of 8 μm were used to establish an indirect coculture system for transfected NK cells and HTR-8/SVneo cells. 1×10^5^ HTR-8/SVneo cells were inoculated in a 200 µL of medium free of FBS in the upper compartment, and transfected NK cells (2×10^5^/mL) were inoculated in a 1 mL medium containing 10% FBS in the lower compartment. For invasion experiments, a matrix gel was used (50 µL), but not for migration experiments. After coculturing at 37°C, 5% CO_2_ overnight, the upper chamber was removed, and the migrated or invasive cells were fixed with formaldehyde, stained with crystal violet, and finally viewed under a microscope. In the rescue experiment, the human IFN-γ blocking antibody (10 μg/mL, 502509, BioLegend, USA) was added to the medium.

### Western blot analysis

Proteins were extracted from DICs and NK cells using RIPA lysis buffer (Beyotime, Shanghai, China) supplemented with 1% PMSF (Beyotime, Shanghai, China), after which the protein concentration was detected using a BCA Protein Assay Kit (Beyotime, Shanghai, China). The protein samples were resolved via sodium dodecyl sulfate-polyacrylamide gel electrophoresis (SDS‒PAGE) and electroblotted onto polyvinylidene difluoride membranes (Millipore, USA). The blotted membrane was blocked with blocking buffer (Beyotime Shanghai, China) for 1 h at room temperature, followed by incubation with primary antibodies at 4°C overnight. The primary antibodies used were as follows: MMP2 (A19080; ABclonal, Wuhan, China), MMP9 (ET1704-69; Huabio, Hangzhou, China), T-bet (13700-1-AP; ProteinTech, Wuhan, China), IFN-γ (15365-1-AP; ProteinTech, Wuhan, China), and GAPDH (60004-1-Ig; ProteinTech, Wuhan, China). Later, the blotted membranes were rinsed in TBST buffer and then incubated with the secondary antibody HRP-conjugated anti-rabbit/mouse IgG for 1 h. Finally, the bands were detected by an enhanced ECL chemiluminescent substrate kit (Beyotime Shanghai, China), and the relative fluorescence intensity was quantified using ImageJ software.

### Statistical analysis

The statistical analysis was performed with GraphPad Prism version 8.0 (GraphPad Software, USA). First, the normality of the distribution of the data was evaluated. The results are expressed as the mean ± S.E.M. Intergroup differences were examined by either unpaired t-tests or the Mann-Whitney U test based on the normality test, and differences were considered significant when the *P* value < 0.05.

## Results

### miR-122-5p is downregulated in dNK cells of patients with URPL

**Figure 1:**
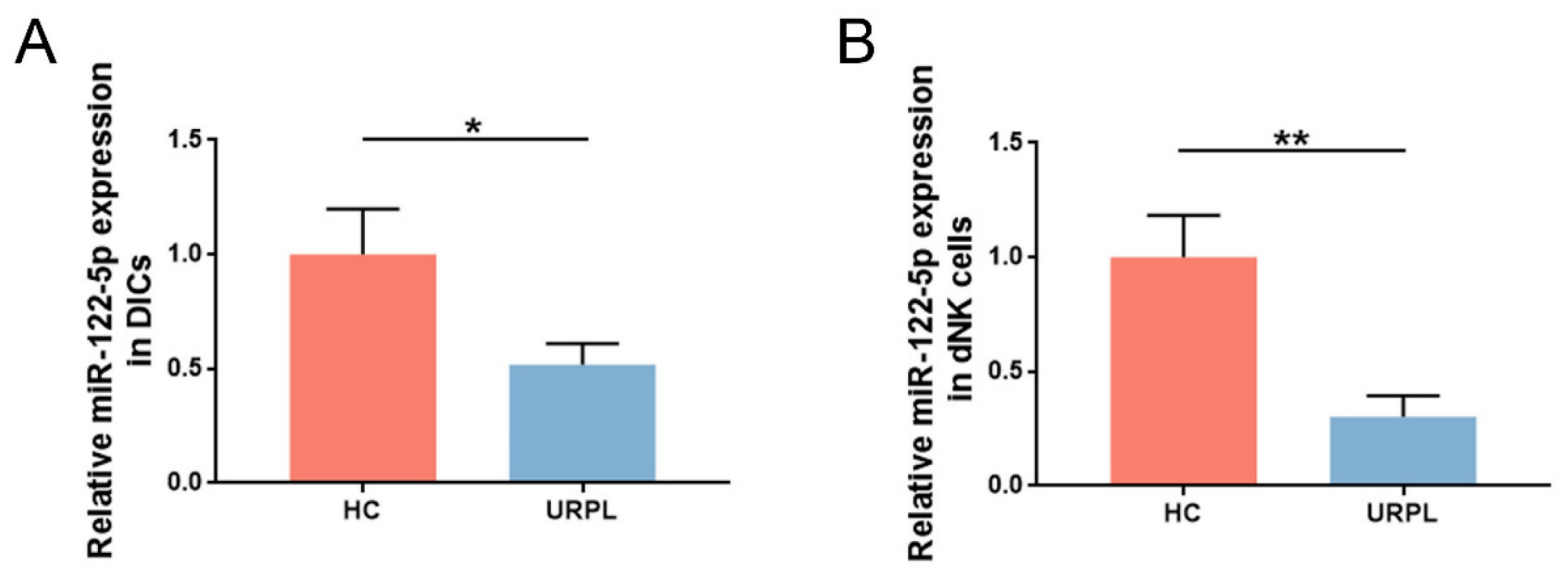
** miR-122-5p expression in dNK cells was determined in the HC group and the URPL group.** (A) RT**-**qPCR was performed to determine the difference in miR-122-5p expression in DICs between the HC group (n = 10) and the URPL group (n = 10). *P< 0.05. (B) RT**-**qPCR was performed to determine the difference in miR-122-5p expression in dNK cells between the HC and URPL groups (n = 5). **P< 0.01. HC, healthy control. URPL, unexplained recurrent pregnancy loss.

Earlier RNA-sequencing results (NCBI Bioproject ID: PRJNA813430) showed that miR-122-5p expression in DICs was lower in patients with URPL than in those with normal pregnancy 16. DICs and dNK cells were extracted from the decidua, and miR-122-5p expression was measured using RT-qPCR. Comparative studies were aligned with sequencing findings (*P*= 0.04; Figure 1A). Considering the high frequency of dNK cells at the maternal-fetal interface in the early stages of pregnancy, miR-122-5p expression in dNK cells from the URPL and HC groups was examined. A significant reduction in miR-122-5p expression was observed in the URPL group compared with the HC group (*P*= 0.009; Figure 1B), indicating that the miR-122-5p deficit in dNK cells may play a role in the URPL development.

### miR-122-5p overexpression or knockdown in NK cells

**Figure 2:**
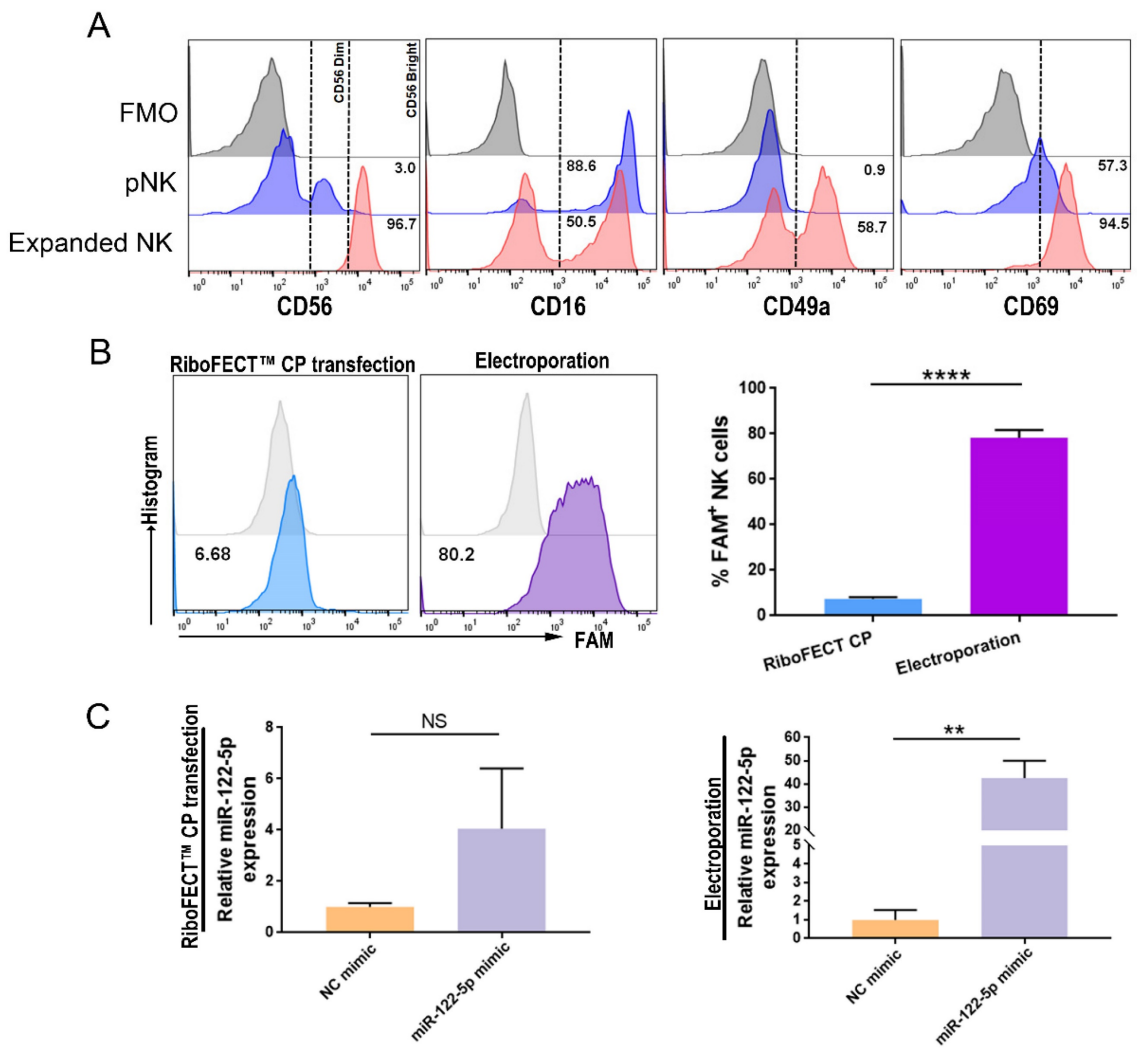
** miR-122-5p overexpression and knockdown in expanded NK cells.** (A) The percentage of CD56^bright^+, CD16+, CD49a+, and CD69+ expanded NK cells were analyzed via flow cytometry. (B) The efficiency of RiboFECT^TM^ and electroporation transfection were determined by flow cytometry (n = 4). ****P< 0.0001. (C) RT**-**qPCR was performed to determine the miR-122-5p expression in transfected NK cells after treatment with the miR-122-5p mimic or NC mimic (n = 3). **P< 0.01. pNK, peripheral natural killer cell. NC, negative control. NS, no significance. FMO, fluorescence minus one.

Grown NK cells had a phenotype similar to that of decidua, which was characterized by higher expression of the activation marker CD69 and the resident marker CD49a (Figure 2A). The transfection efficacy of RiboFECT™ CP reagent-driven transfection and electroporation was assessed using flow cytometry (*P*< 0.0001; Figure 2B). Results indicated that electroporation of a miR-122-5p mimic resulted in an approximately 48-fold elevation in the miR-122-5p expression in NK cells (*P*= 0.26; *P*= 0.005; Figure 2C).

### Regulation of activation and apoptosis of NK cells by miR-122-5p

**Figure 3:**
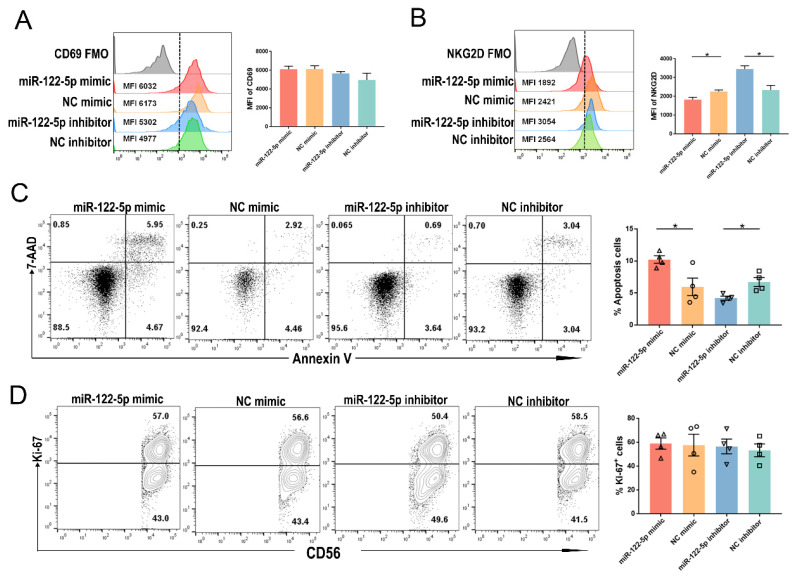
** miR-122-5p is involved in the regulation of NK cell activation and apoptosis.** (A) The MFI of CD69 in NK cells transfected with miR-122-5p mimic and inhibitor treatment was determined by flow cytometry (n = 3). (B) The MFI of NKG2D in NK cells treated with miR-122-5p mimic and inhibitor treatment was determined by flow cytometry (n = 3) *P< 0.05. (C) Effect of miR-122-5p mimic and inhibitor on the percentage of apoptotic NK cells detected by flow cytometry. *P< 0.05. (D) Effect of miR-122-5p mimic and inhibitor on the proliferation potential of NK cells, as detected by flow cytometry. NC, negative control. FMO, fluorescence minus one. MFI, mean fluorescence intensity.

The URPL group showed a higher percentage of CD69+ NK cells than the HC group (*P*= 0.03; Supplementary Figure S2A). The experiment involved introducing a miR-122-5p mimic (inhibitor) or control into cells to investigate the impact of miR-122-5p on the biological functions of NK cells. NK cells transfected with miR-122-5p inhibitor (mimic) exhibited a higher (lower) mean fluorescence intensity (MFI) of NKG2D compared to the control group (*P*= 0.02; *P*= 0.04). However, no significant changes were observed in the MFI of CD69 between the mimic (inhibitor) and control groups (*P*= 0.39; *P*= 0.94; Figure 3A and 3B). Flow cytometric analysis revealed an increased (decreased) apoptosis rate in NK cells in the mimic (inhibitor) group compared to the control group (*P*= 0.02; *P*= 0.01; Figure 3C). Nevertheless, there was no significant difference in the percentage of Ki-67+ NK cell proliferation potential across the four groups with varying miR-122-5p expression (*P*= 0.90; *P*= 0.70; Figure 3D).

### miR-122-5p regulates inflammation and cytotoxicity in NK cells

**Figure 4:**
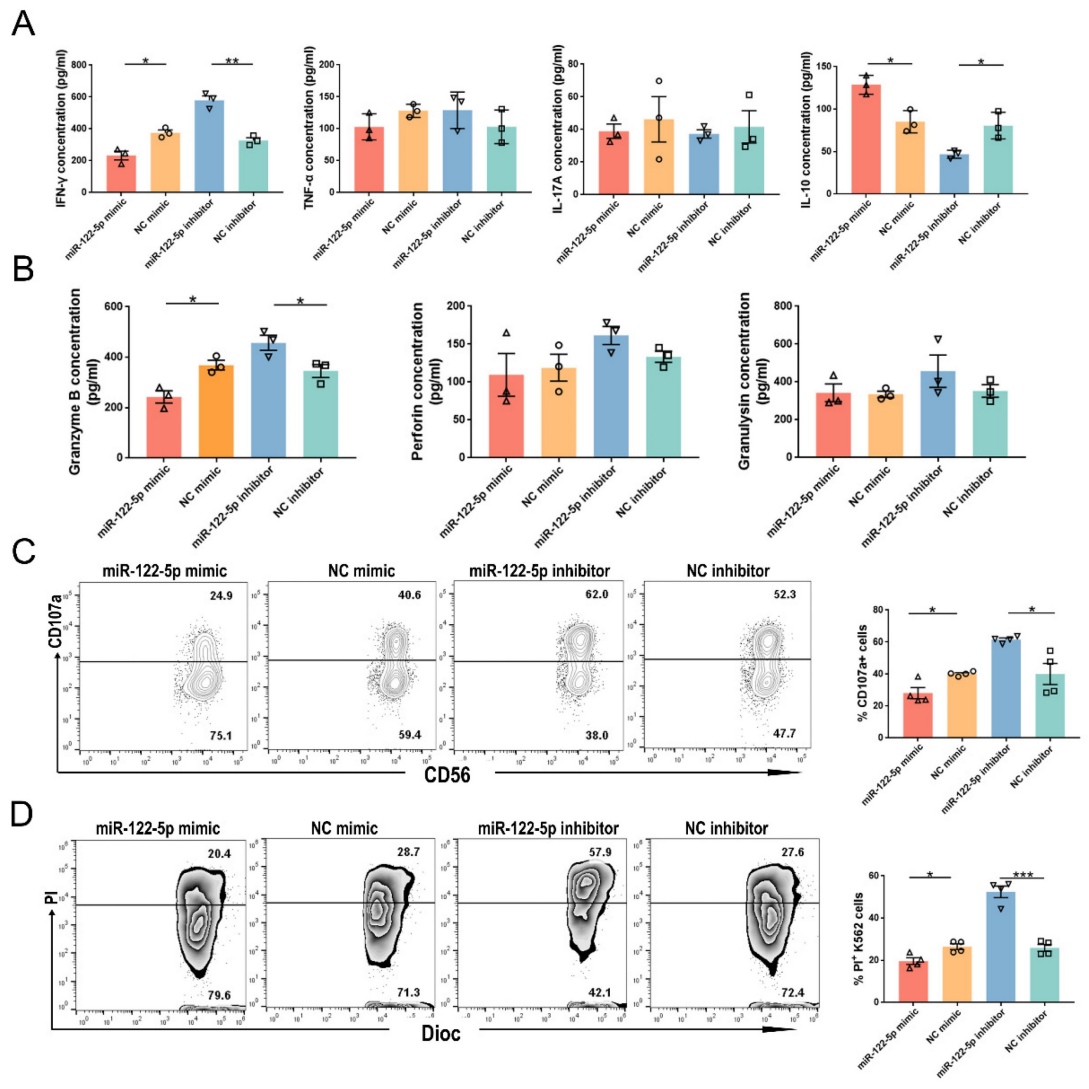
** miR-122-5p is involved in the immune function regulation of NK cells.** (A) Effect of miR-122-5p mimic and inhibitor on the secretion of inflammatory factors by NK cells, as detected by flow cytometry. *P< 0.05, **P< 0.01. (B) Effect of miR-122-5p mimic and inhibitor on the secretion of lytic granules by NK cells, as detected by ELISA. *P< 0.05. (C) Effect of miR-122-5p mimic and inhibitor on the degranulation of NK cells detected by flow cytometry. *P< 0.05. (D) Effect of miR-122-5p mimic and inhibitor on the killing ability of NK cells, as detected by flow cytometry. *P< 0.05, ***P< 0.001.

The release of inflammatory factors and lytic granules is essential for the proper functioning of dNK cells. Flow cytometry analysis showed that the URPL group had a higher proportion of IFN-γ+ and interleukin (IL)-17A+ NK cells than the HC group (*P*= 0.009; *P*= 0.01; Supplementary Figure S3). The URPL group had a lower frequency of IL-10+ NK cells (*P*= 0.008). The involvement of miR-122-5p in the regulation of cytokine secretion was confirmed using ELISA. After treatment with the miR-122-5p mimic (inhibitor), along with a reduction (elevation) in IFN-γ secretion (*P*= 0.01;* P*= 0.001), there was an elevation (reduction) in IL-10 secretion (*P*= 0.01;* P*= 0.02) by NK cells. However, no discernable IL-17A or TNF-α secretion alterations were detected (*P*> 0.05; Figure 4A).

NK cells treated with the miR-122-5p mimic (inhibitor) showed a reduction (increase) in the granzyme B secretion (*P*= 0.01; *P*= 0.04; Figure 4B). There were no noticeable disparities in the perforin or granulysin production (*P*> 0.05). Alteration of lytic granules corresponded to the variation in dNK cells of patients with URPL (Supplementary Figure S4). Ultimately, NK cell cytotoxicity was observed after treatment with miR-122-5p mimic or inhibitor. The percentages of CD107a+ NK cells (*P*= 0.01; *P*= 0.01) and PI+ K562 cells (*P*= 0.01; *P*= 0.0002) declined (increased) in the miR-122-5p mimic (inhibitor) transfection group after effective cell-target cell coculture (Figure 4C and 4D).

### miR-122-5p regulates IFN-γ in NK cells by targeting *T-bet*

**Figure 5:**
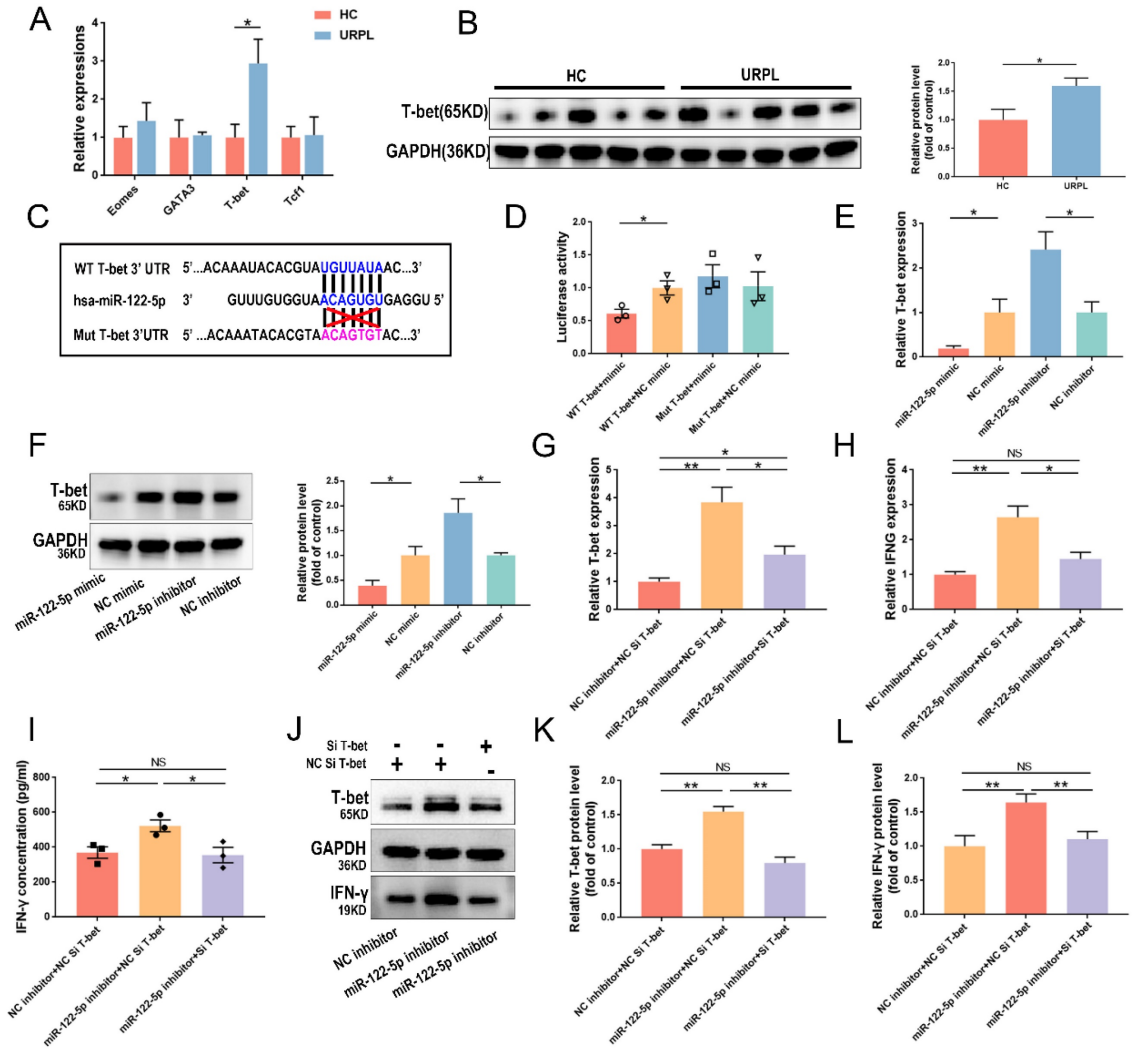
** Verification of the miR-122-5p/T-bet/IFN-γ axis in NK cells.** (A)* T-bet*, *Eomes*,* GATA3*, and* Tcf1* expression in decidual NK cells was compared between the HCs and URPL patients (n = 5). *P< 0.05. (B) The protein expression of T-bet in decidual NK cells was compared between the HC group and the URPL group (n = 5). *P< 0.05. (C-D) The binding site between *T-bet* and miR-122-5p, as predicted by the *RNAhybrid* database was detected via a luciferase reporter assay. *P< 0.05. (E-F) RT-qPCR and Western blot analysis of the effects of miR-122-5p mimic and inhibitor on* T-bet* mRNA and protein levels in NK cells. *P< 0.05 (G-I) RT-qPCR and ELISA analysis of the effects of cotransfection of miR-122-5p inhibitor and *T-bet* siRNA on the mRNA expression of *T-bet* and *IFNG* and the secretion of IFN-γ in NK cells (n = 4; n = 3). *P< 0.05, **P< 0.01. (J-L) Western blot analysis of the effects of co-transfection of NK cells with miR-122-5p inhibitor and *T-bet* siRNA on the protein expression of T-bet and IFN-γ (n = 4). *P< 0.05, **P< 0.01. HC, healthy control. URPL, unexplained recurrent pregnancy. NS, no significance.

*T-bet*, *Eomes*, *GATA3*, and *Tcf1* were selected as target genes as predicted by the* RNAhybrid* database. The upregulation of *T-bet* mRNA expression in dNK cells from patients with URPL was verified using RT-qPCR (*P*= 0.02; Figure 5A). The T-bet protein level increased significantly in patients with URPL, and it was inversely related to miR-122-5p expression in dNK cells (*P*= 0.03; Figure 5B). To understand this further, wild-type and mutant plasmid vectors for *T-bet* were created using the sequence predicted by *RNAhybrid* database (Figure 5C). The dual-luciferase reporter experiment provided evidence of the interaction between miR-122-5p and *T-bet* (Figure 5D). Results of subsequent experiments showed that miR-122-5p can negatively regulate the mRNA (*P*= 0.03; *P*= 0.02) and protein expression of *T-bet*. (*P*= 0.04; *P*= 0.03) (Figure 5E and 5F).

Efficacy of *T-bet* silencing and its influence on IFN-γ in NK cells following *T-bet* siRNA treatment was evaluated (*P*< 0.05). *T-bet* siRNA-transfected NK cells showed reduction in IFN-γ production (*P*< 0.01) (Supplementary Figure S5A and S5B). Cotransfection of NK cells with a miR-122-5p inhibitor and *T-bet* siRNA was performed to investigate potential cascading relationships among miR-122-5p, *T-bet*, and IFN-γ. Results showed that* T-bet* siRNA reversed the increase in mRNA expression (Figure 5G and 5H) of *T-bet* and* IFNG* in transfected NK cells with miR-122-5p inhibitor and secretion of IFN-γ (Figure 5I). Further rescue experiments confirmed the presence of a miR-122-5p/T-bet/IFN-γ regulatory axis in NK cells (Figure 5J-5L).

### Changes in miR-122-5p expression in NK cells affect the biological behavior of trophoblasts

**Figure 6:**
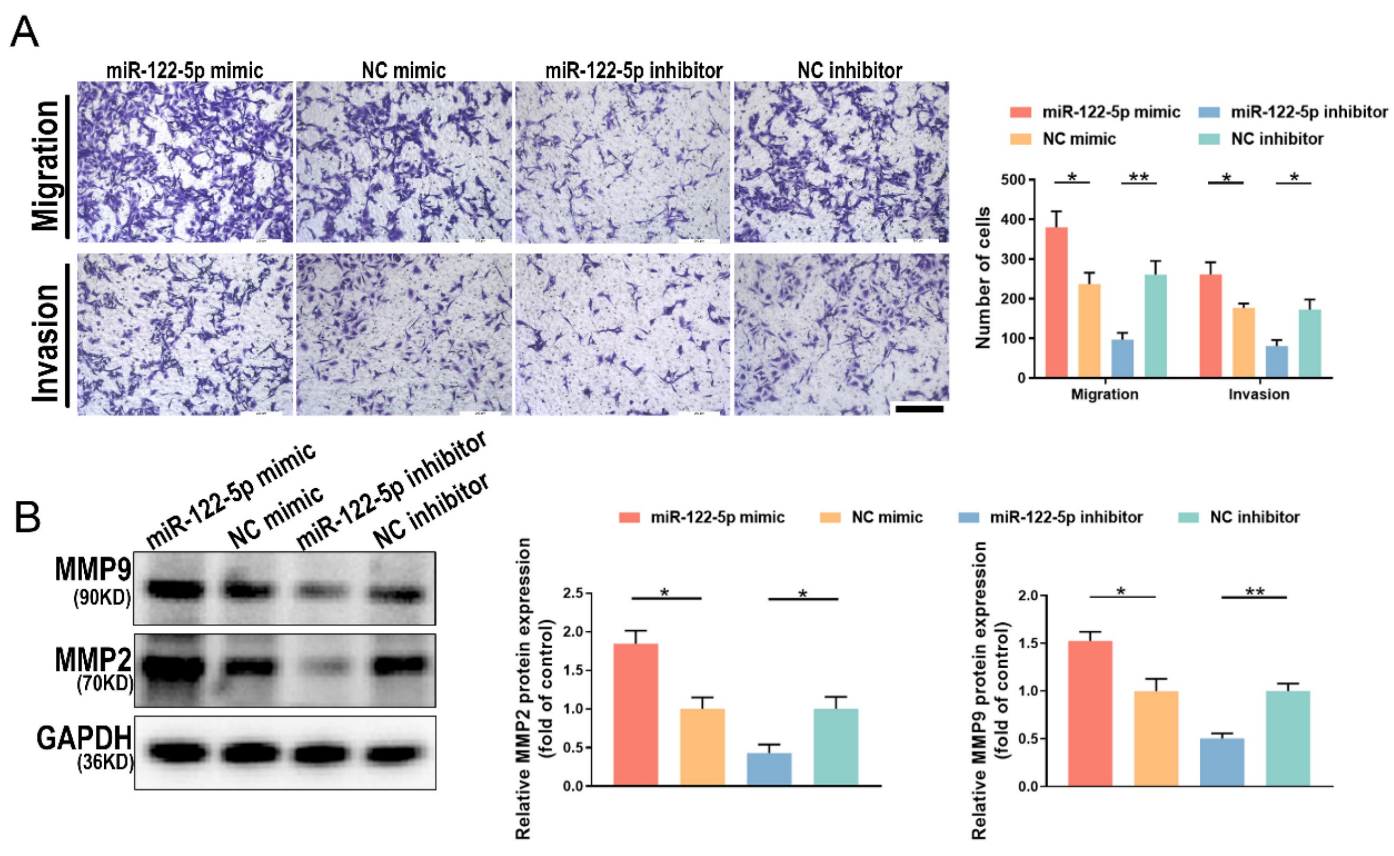
** Altered miR-122-5p expression in NK cells affects the biological function of HTR-8/SVneo cells.** (A) Effect of miR-122-5p mimic and inhibitor in NK cells on the migration and invasion of HTR-8/SVneo cells detected by coculture without or with matrigel (n = 4). The scale bar represents 200 μm. *P< 0.05, **P< 0.01. (B) Effect of miR-122-5p mimic and inhibitor in NK cells on the protein levels of MMP2 and MMP9 in HTR-8/SVneo cells detected by coculture without or with matrigel (n = 3). *P< 0.05, **P< 0.01.

For a successful pregnancy, the appropriate development of the spiral artery and trophoblast cell invasion requires good communication between the fetus and mother. To investigate the impact of NK cell alteration of miR-122-5p expression on embryonic development, NK cells were cultured alongside HTR-8/SVneo cells after exposure to a miR-122-5p mimic or inhibitor. Results demonstrated that the quantity of migrating and invasive HTR-8/SVneo cells in the miR-122-5p mimic group increased (*P*= 0.02; *P*= 0.04), whereas the group treated with an inhibitor decreased (*P*= 0.005; *P*= 0.01; Figure 6A). Matrix metalloproteinase (MMP) 2 and 9 belong to the MMP family and are essential enzymes that break down the basement membrane. Additional experiments were conducted to quantify the protein expression levels of MMP2 and MMP9 in different treatment groups of HTR-8/SVneo cells (Figure 6B). Results revealed a correlation between fluctuations in the expression levels of MMP2 (*P*= 0.04; *P*= 0.04) and MMP9 (*P*= 0.03; *P*= 0.005) and the alterations in migration and invasion numbers, strengthening our initial findings.

### miR-122-5p in NK cells affects trophoblasts via the miR-122-5p/T-bet/IFN-γ axis

**Figure 7:**
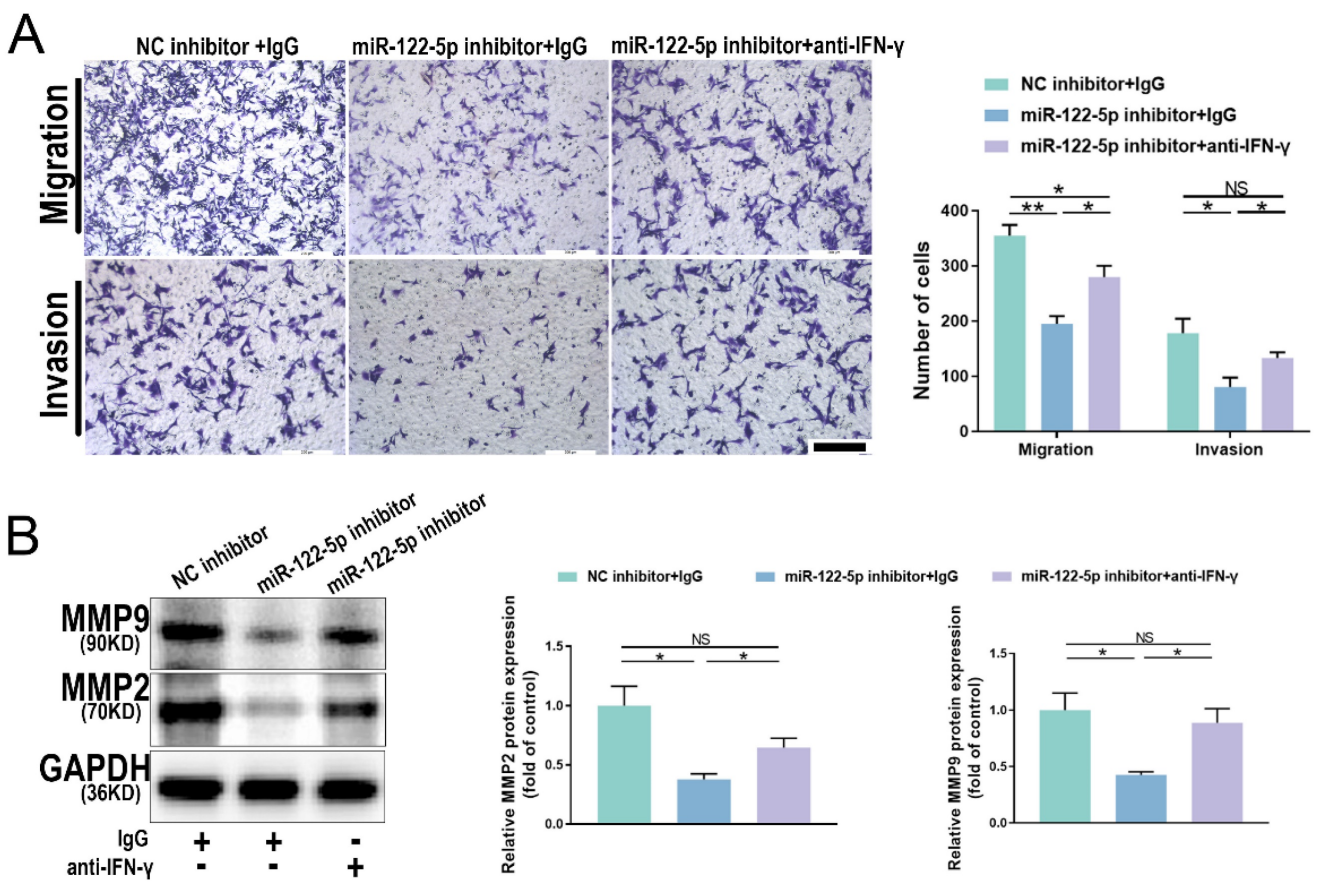
** IFN-γ alterations affect the biological function of HTR-8/SVneo cells.** (A) Effect of miR-122-5p inhibitor in NK cells on the migration and invasion of HTR-8/SVneo cells detected by coculture without or with the addition of anti-IFN-γ (n = 4). The scale bar represents 200 μm. *P< 0.05, **P< 0.01. (B) Effect of miR-122-5p inhibitor in NK cells on the protein levels of MMP2 and MMP9 of HTR-8/SVneo cells detected by coculture without or with the addition of anti-IFN-γ (n = 3). *P< 0.05. NS, no significance.

The proportion of IFN-γ+ NK cells was increased in patients with URPL, and NK cells with reduced miR-122-5p expression released more IFN-γ. To understand the molecular mechanisms by which IFN-γ affects trophoblast migration and invasion, NK cells treated with a miR-122-5p inhibitor were cultured with a neutralizing antibody against IFN-γ. Results indicated that the combined use of the miR-122-5p inhibitor and neutralizing antibody effectively restored the migratory and invasive abilities of trophoblast cells, surpassing those of the control group (*P*= 0.01; *P*= 0.03; Figure 7A). Similarly, the protein levels for MMP2 and MMP9 noticeably increased (*P*= 0.04; *P*= 0.02; Figure 7B). This analysis showed that the miR-122-5p/T-bet/IFN-γ axis plays a crucial role in maternal-fetal interactions by controlling the expression of invasion-related proteins, which affects the biological activity of trophoblast cells.

## Discussion

**Figure 8:**
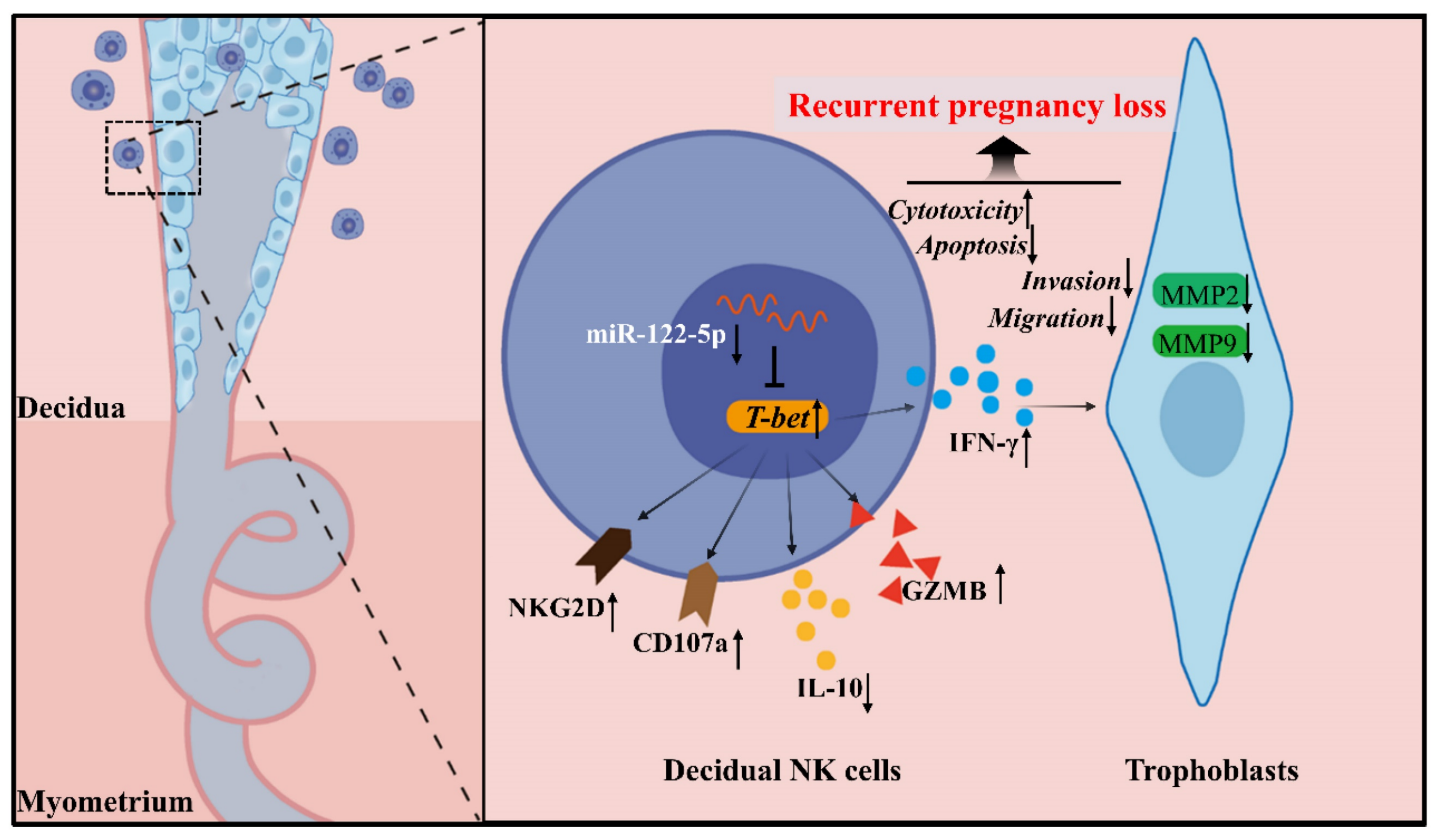
** Schematic diagram of the action of miR-122-5p signaling in dNK cells at the maternal-fetal interface in patients with URPL.** The decreased miR-122-5p expression in decidual natural killer cells may lead to reduced apoptosis, heightened activation, and immunological dysregulation. Moreover, it indirectly affects the normal biological behavior of trophoblast cells by targeting *T-bet* and subsequent IFN-γ secretion, ultimately resulting in the occurrence of recurrent pregnancy loss. GZMB, granzyme B.

The mechanisms governing the biological and immunological functions of dNK cells, an essential component of the immune microenvironment at the maternal-fetal interface that upholds immune tolerance and balance, are diverse and complex. This study revealed a noteworthy miR-122-5p downregulation in dNK cells, which is implicated in URPL. miR-122-5p regulates NK cell apoptosis and immune activity. This study supported the existence of the miR-122-5p/T-bet/IFN-γ regulatory axis within NK cells, which may affect the biological functions of trophoblast cells (Figure 8).

Studies on noncoding RNAs at the maternal-fetal interface have mostly focused on regulating trophoblasts and decidual stromal cell activities 17, 18. The immunological mechanisms underlying RPL remain unknown, and it is difficult to transfect primary immune cells from tissue in a durable and successful manner 19. According to Du *et al.*'s study 20, the expansion of NK cells with a resident phenotype was induced to partially substitute for dNK cells. Our data suggest that miR-122-5p impairs dNK cell immunological function, compromising immune tolerance at the maternal-fetal interface and hindering embryo colonization.

Adverse inflammatory changes in locally infiltrating immune cells will lead to the occurrence of the disease 21, 22. The balance of inflammatory factors at the maternal-fetal interface may be upset by decreased IL-10 secretion, whereas decreased miR-122-5p expression causes NK cells to secrete more IFN-γ. The cytotoxic properties of NK cells are strictly regulated by hormonal regulation and receptors such as CD49a, Tim-3, and NKG2A 23-25. Granzyme B release in excess may compromise the immunological tolerance of developing embryos 26. A previous study demonstrated that miR-30e targets* PRF1* to maintain immunological tolerance 27. miR-122-5p is a significant modulator of lipid metabolism and is widely expressed in the mature liver, where it plays a role in the pathophysiology of nonalcoholic steatohepatitis and HCC 28, 29. Studies have also shown that miR-122-5p mediates cerebral ischemic injury and acute myocardial infarction 30, 31. This study reported the function of miR-122-5p at the maternal-fetal interface for the first time and found that it mediates URPL occurrence.

The transcription factors Eomes, KLF2, and Elf1 govern NK cell development and maturation 32, 33. This study observed an increase in the Th1 transcription factor T-bet in URPL-derived dNK cells; this increase is controlled negatively by miR-122-5p. As demonstrated in earlier studies, *T-bet* had increased mRNA expression in the decidua of patients with preeclampsia 34. Normally, CD56^bright^ NK cells show modest levels of *T-bet* mRNA expression, and a significant increase suggests an aberrant functional difference among NK cells 35. Based on these supporting investigations, *T-bet* is a prominent molecule responsible for regulating IFN-γ through miR-122-5p. Therefore, the miR-122-5p/T-bet/IFN-γ regulation pathway in NK cells may significantly enhance maternal-fetal tolerance.

Abnormal invasion of trophoblast cells can lead to complications such as preeclampsia and intrauterine growth restriction, and trophoblast cells employ MMPs and urokinase activator systems to degrade the basement membrane and extracellular matrix 36, 37. Immune cells and vascular formation, remodeling, and calcification are strongly associated, with the placenta being a highly vascularized tissue 38. The reciprocal interaction between trophoblasts and dNK cells seems to limit and augment each other, creating the necessary equilibrium for the continuation of the fetus 39, 40. In this study, we observed a reduced invasive capacity of HTR-8/ SVneo cells due to reduced miR-122-5p expression, leading to excessive IFN-γ secretion. Verma *et al.* showed that IFN-γ inhibited STAT1 and AKT pathways by upregulating the expression of bone marrow stromal antigen 2 (BST-2) and E-cadherin, thereby curtailing the invasive ability of trophoblast cells 41. To understand the molecular mechanism responsible for IFN-γ inhibition, an IFN-γ neutralizing antibody was administered to the group exhibiting reduced miR-122-5p expression. There was a partial recovery of the decreased invasion of HTR-8/SVneo cells accompanied by reduced production of MMP2 and MMP9 protein. miR-122-p in dNK cells regulates IFN-γ production, affecting the expression of functional proteins relevant to trophoblasts.

Embryo colonization and development occur in hypoxic conditions 42. Previous studies have reported that mitochondrial function is closely related to *T-bet* expression in NK cells and its anti-tumor activity 43. The anomalous metabolism and function of immune cells in specific environments affect blood perfusion, hyperactivation, and inflammation 44, 45. Researchers have found that targeted mitochondrial correction improves the ischemic state and stress of myocardial vessels, providing new insights for the future treatment of ischemic placental disease 46, 47. Recent studies have reported an updated bioswitchable delivery system for achieving controlled delivery based on tetrahedral DNA nanostructures and microRNA loading and release. This suggests the possibility of clinical application of intervention with abnormal miR-122-5p expression 48. The limitation of this study was that transfection of primary dNK cells was not achieved. Additional *in vivo* studies are warranted to further corroborate our findings and the impact of miR-122-5p on the entire immune microenvironment at maternal-fetal interface should be explored in the future.

## Conclusion

In summary, this study revealed that miR-122-5p plays a crucial role in controlling NK cell activity. Abnormal miR-122-5p expression in dNK cells indirectly affects the biological function of trophoblasts at the maternal-fetal interface, which is implicated in URPL occurrence. Hence, specifically regulating miR-122-5p/T-bet/IFN-γ axis in dNK cells is a prospective therapeutic approach for URPL.

## Supplementary Material

Supplementary figures and table.

## Figures and Tables

**Table 1:** List of antibodies.

Antibodies	Fluorochrome	Clone	Dilution	Sources
anti-CD3	FITC	UCHT1	1:100	BioLegend
anti-CD45	APC-cy7	HI30	1:100	BioLegend
anti-CD49a	APC	TS2/7	1:100	BioLegend
anti-CD56	PE-cy7	HCD56	1:100	B&D
anti-CD69	FITC	FN50	1:100	BioLegend
anti-CD16	BV421	3GB	1:100	BioLegend
anti-NKG2D	BV605	1D11	1:100	BioLegend
anti-Granzyme B	BV421	QA18A28	1:50	BioLegend
anti-Perforin	FITC	dG9	1:50	BioLegend
anti-Granulysin	APC	DH2	1:50	BioLegend
anti-TNF-α	APC	MAb11	1:50	BioLegend
anti-IFN-γ	FITC	B27	1:50	BioLegend
anti-IL17A	BV605	BL168	1:50	BioLegend
anti-IL10	BV421	JES3-9D7	1:50	BioLegend
anti-CD107a	BV421	H4A3	1:50	BioLegend
anti-Ki67	BV421	Ki-67	1:50	BioLegend
anti-7AAD	FITC	-	1:100	BioLegend
Annexin V	PE	-	1:100	BioLegend
Propidium Iodide	PE	-	1:100	Thermo Fisher
Dioc	FITC	-	1:500	Thermo Fisher
Zombie Aqua^TM^	BV510	-	1:500	BioLegend

**Table 2:** RT-qPCR primers

Primer	Sequence
GAPDH-F	5'-ATGACATCAAGAAGGTGGTG-3'
GAPDH-R	5'-CATACCAGGAAATGAGCTTG-3'
TBX21-F	5'-AACCACCTGTTGTGGTCCAA-3'
TBX21-R	5'-CCCGGCCACAGTAAATGACA-3'
IFNG-F	5'-AGTGATGGCTGAACTGTCGC-3'
IFNG-R	5'-CTGGGATGCTCTTCGACCTC-3'
EOMES-F	5'-TCACCAATAACAAAGGCGCAAAT-3'
EOMES-R	5'-CACGCCATCCTCTGTAACTTCAA-3'
Tcf1-F	5'-CACTCCCATGAAGACGCAGAAG-3'
Tcf1-R	5'-CCTTCTTGGTTGGTAGCTCATCA-3'
GATA3-F	5'-GTGAACTGTGGGGCAACCTC-3'
GATA3-R	5'-GGTCTGACAGTTCGCACAGG-3'

## Abbreviations

URPL: Unexplained recurrent pregnancy loss
HC: Healthy control
DICs: Decidual immune cells
dNK: Decidual natural killer cells
GZMB: granzyme B
CD: Cluster of differentiation
hCG: Human chorionic gonadotropin
IL: Interleukin
FMO: Fluorescence minus one
MFI: Mean fluorescence intensity
mRNA: Message RNA
RT-qPCR: Reverse transcription-polymerase chain reaction. ELISA, Enzyme-linked immunosorbent assay
