# Supplementary material for: Abnormal miR-122-5p expression in decidual NK cells and its impact on trophoblast behavior: insights into unexplained recurrent pregnancy loss
Source: Int J Med Sci. 2024 Oct 28;21(14):2824–36. doi: 10.7150/ijms.101865 (PMC11539378; doi:10.7150/ijms.101865)
Supplement: Supplementary file 1 — Supplementary figures and table. [file ijmsv21p2824s1.pdf]

Supplementary Information

Title: Abnormal miR-122-5p expression in decidual NK cells and its impact on trophoblast behavior: insights into unexplained recurrent pregnancy loss

Authors: Yuan Liu, Liman Li, Ting Feng, Wenjie Zhou, Ying Liu, Yueli Mu, Zhuoxu He and Hong Li *

Contents:

Supplementary Table S1. Demographic data of the HC group and the URPL group

Supplementary Figure S1. The gating strategy of decidual NK cells.

Supplementary Figure S2. Comparison of the expression of CD69 and NKG2D in dNK cells of the HC group and the URPL group

Supplementary Figure S3. Comparison of the expression of IFN-γ, TNF-α, IL-17A, and IL-10 in dNK cells of the HC group and the URPL group

Supplementary Figure S4. Comparison of the expression of granzyme B, perforin, and granulysin in dNK cells of the HC group and the URPL group

Supplementary Figure S5. Verification of the knockdown efficiency of *T-bet*.

Supplementary Figure S6. Full western blots for Figure 5.

Supplementary Figure S7. Full western blots for Figure 6.

Supplementary Figure S8. Full western blots for Figure 7.

Supplementary Figure S9. Full western blots for Supplementary Figure S5.

Supplementary Table S1 Demographic data of the HC group and the URPL group

	HC（n = 13）	URPL（n = 15）	*P*
Maternal age (years)	29.92 ± 1.26	30.9 ± 1.97	0.67
Gestational age (weeks)	8.95 ± 0.18	9.1 ± 0.21	0.51
BMI (kg/m^2)	23.16 ± 0.51	22.42 ± 0.42	0.27
Gravidity	2.15 ± 0.22	2.40 ± 0.21	0.43
Parity	1.15(0.67,1.64)	0(0,0)	<0.0001

BMI, body mass index.

Supplementary Figures

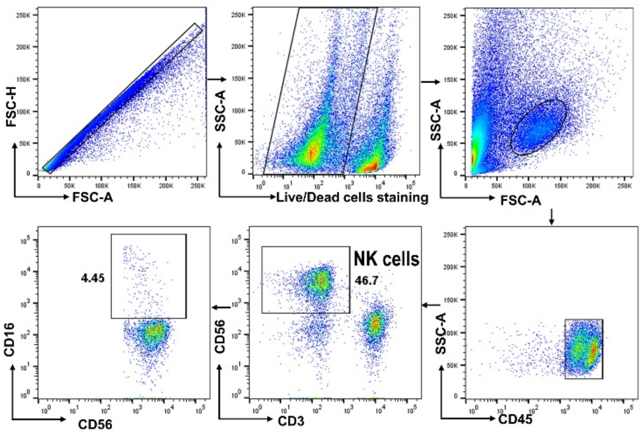

Supplementary Figure S1. The gating strategy of decidual NK cells

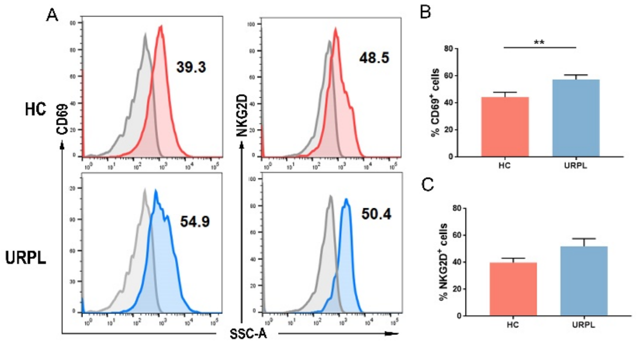

Supplementary Figure S2. Comparison of the expression of CD69 and NKG2D in dNK cells of the HC group and the URPL group

(A-C) Flow cytometry determined the percentage of CD69+ and NKG2D+ NK cells in the HC group (n = 13) and URPL group (n = 15). HC, healthy control. URPL, unexplained recurrent pregnancy. ***P*< 0.01.

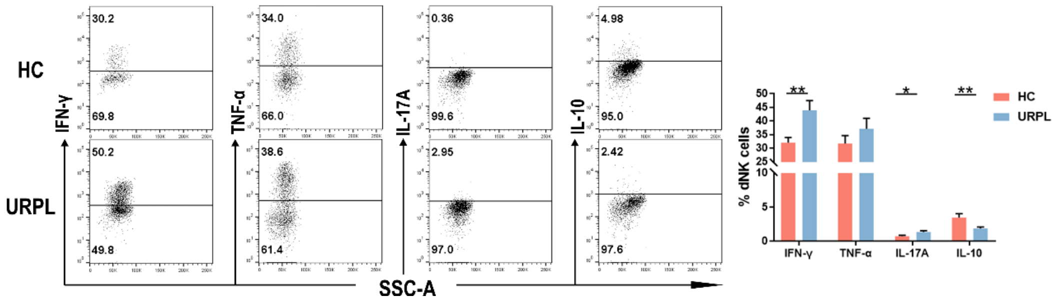

Supplementary Figure S3. Comparison of the expression of IFN-γ, TNF-α, IL-17A, and IL-10 in dNK cells of the HC group and the URPL group

Flow cytometry determined the percentage of IFN-γ+, TNF-α+, IL-17A+, and IL-10+ NK cells in the HC group (n = 13) and URPL group (n = 15). HC, healthy control. URPL, unexplained recurrent pregnancy. **P*< 0.05, ***P*< 0.01.

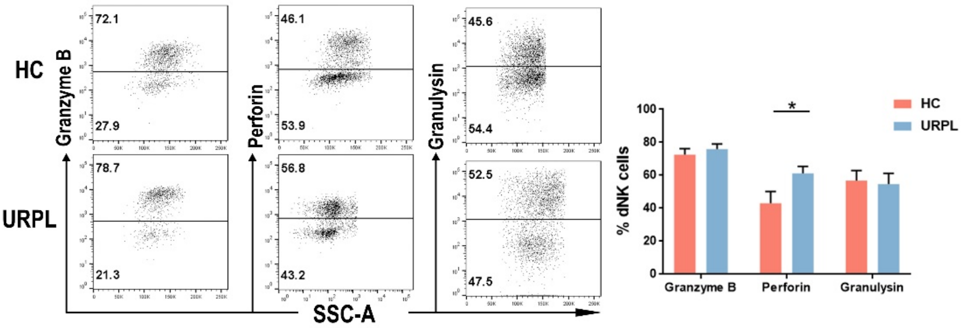

Supplementary Figure S4. Comparison of the expression of granzyme B, perforin, and granulysin in dNK cells of the HC group and the URPL group

Flow cytometry determined the percentage of granzyme B+, perforin+, and granulysin+ NK cells in the HC group (n = 13) and URPL group (n = 15). HC, healthy control. URPL, unexplained recurrent pregnancy. **P*< 0.05.

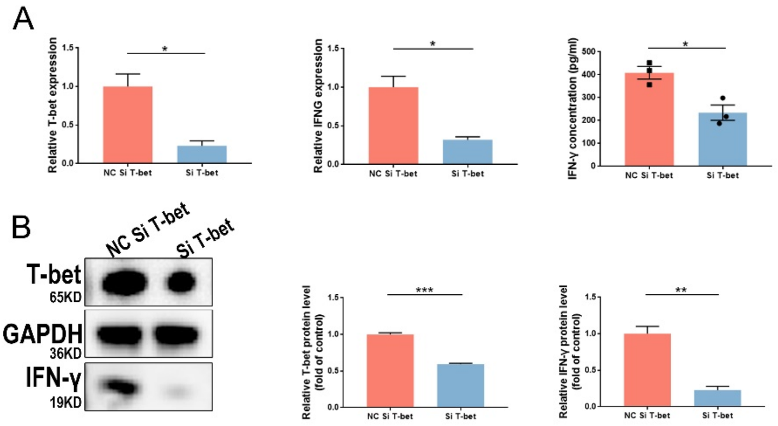

Supplementary Figure S5. Verification of the knockdown efficiency of *T-bet*

(A) RT-qPCR and ELISA analysis of the effects of NC and *T-bet* siRNA on mRNA

expression of *T-bet* and *IFNG*, secretion of IFN-γ in NK cells (n = 3). *P< 0.05. (B)Western blot analysis of the effects of NC and *T-bet* siRNA on the protein of T-bet and IFN-γ in NK cells (n = 3). *P< 0.05, **P< 0.01, ***P< 0.001. HC, healthy control. URPL, unexplained recurrent pregnancy.

Figure 5B

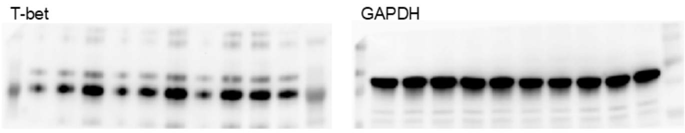

Figure 5F

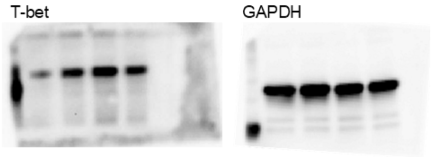

Figure 5J

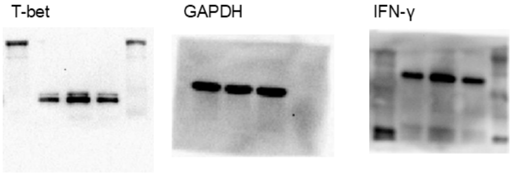

Supplementary Figure S6. Full western blots for Figure 5

Uncropped western blots of those shown in Figure 5.

Figure 6B

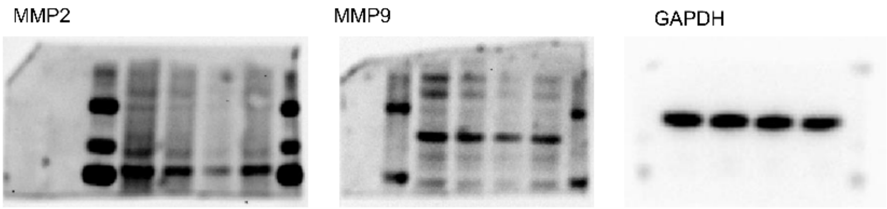

Supplementary Figure S7. Full western blots for Figure 6

Uncropped western blots of those shown in Figure 6.

Figure 7B

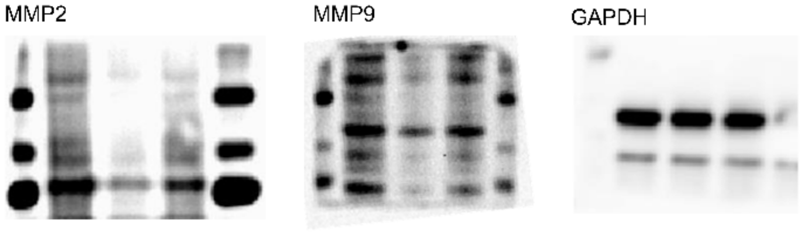

Supplementary Figure S8. Full western blots for Figure 7

Uncropped western blots of those shown in Figure 7.

Figure S5B

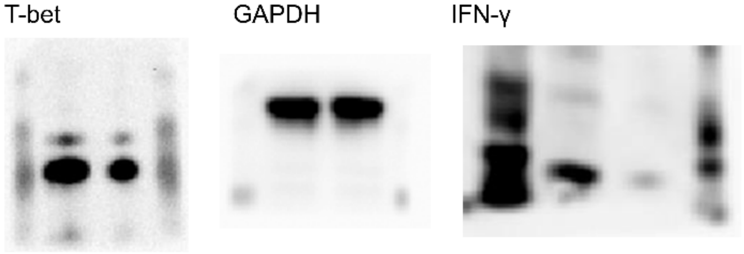

Supplementary Figure S9. Full western blots for Supplementary Figure S5

Uncropped western blots of those shown in Figure S5.
